# Substitutions for arginine at position 780 in triple helical domain of the α1(I) chain alter folding of the type I procollagen molecule and cause osteogenesis imperfecta

**DOI:** 10.1371/journal.pone.0200264

**Published:** 2018-07-10

**Authors:** Elena Makareeva, Guoli Sun, Lynn S. Mirigian, Edward L. Mertz, Juan C. Vera, Nydea A. Espinoza, Kathleen Yang, Diana Chen, Teri E. Klein, Peter H. Byers, Sergey Leikin

**Affiliations:** 1 Section on Physical Biochemistry, National Institute of Child Health and Human Development, National Institutes of Health, Bethesda, Maryland, United States of America; 2 Department of Pathology, University of Washington, Seattle, Washington, United States of America; 3 Department of Genetics, Stanford University, Palo Alto, California, United States of America; 4 Department of Medicine, Division of Medical Genetics, University of Washington, Seattle, Washington, United States of America; Ecole normale superieure de Lyon, FRANCE

## Abstract

OI is a clinically and genetically heterogeneous disorder characterized by bone fragility. More than 90% of patients are heterozygous for mutations in type I collagen genes, *COL1A1* and *COL1A2*, and a common mutation is substitution for an obligatory glycine in the triple helical Gly-X-Y repeats. Few non-glycine substitutions in the triple helical domain have been reported; most result in Y-position substitutions of arginine by cysteine. Here, we investigated leucine and cysteine substitutions for one Y-position arginine, p.Arg958 (Arg780 in the triple helical domain) of proα1(I) chains that cause mild OI. We compared their effects with two substitutions for glycine located in close proximity. Like substitutions for glycine, those for arginine reduced the denaturation temperature of the whole molecule and caused asymmetric posttranslational overmodification of the chains. Circular dichroism and increased susceptibility to cleavage by MMP1, MMP2 and catalytic domain of MMP1 revealed significant destabilization of the triple helix near the collagenase cleavage site. On a cellular level, we observed slower triple helix folding and intracellular collagen retention, which disturbed the Endoplasmic Reticulum function and affected matrix deposition. Molecular dynamic modeling suggested that Arg780 substitutions disrupt the triple helix structure and folding by eliminating hydrogen bonds of arginine side chains, in addition to preventing HSP47 binding. The pathogenic effects of these non-glycine substitutions in bone are probably caused mostly by procollagen misfolding and its downstream effects.

## Introduction

Osteogenesis imperfecta (OI) is a clinically and genetically heterogeneous disorder characterized by susceptibility to fracture [1]. Besides bone fragility, individuals with OI may present with short stature, bone deformities, dentinogenesis imperfecta, blue sclera, scoliosis, hearing loss, and other connective tissue disorders [2]. Although discoveries of causative mutations in over a dozen different genes have generated considerable excitement and identified new biochemical pathways that contribute to OI [3–9], about 90% of individuals with OI have a mutation in one of the two genes, *COL1A1* and *COL1A2*, that encode the proα1(I) and proα2(I) chains of type I collagen, the major protein of bone [7,10].

*COL1A1* and *COL1A2* mutations lead to OI by altering synthesis, assembly, folding, posttranslational modification, secretion, and structure of collagen. The molecular events in the cell and extracellular matrix (ECM) that translate these effects into bone pathology are incompletely understood. In addition to the events in the ECM, disruption of procollagen folding in the osteoblast endoplasmic reticulum (ER) appears to cause bone pathology by affecting cell differentiation and/or function [6,11–13]. At the same time, deficient secretion and/or incorporation of abnormal collagen molecules into ECM can alter bone matrix assembly, mechanical properties, mineralization, and interactions with various growth factors and cells [14,15].

The majority of mutations identified in individuals with severe or lethal forms of OI result in substitutions for glycine residues in the 338 canonical Gly-X-Y repeats of collagen triple helix [1] of the proα1(I) and proα2(I) chains of type I procollagen. Substitutions of glycine (Gly) by amino acids with charged or branched hydrophobic side chains are particularly disruptive for triple helix structure and folding [16], and generally result in the most severe OI outcomes [15]. While it has become clear that such disruptions might cause malfunction of osteoblasts as well as malfunction of collagen molecules in bone matrix, the underlying biochemical pathways and their roles in bone pathology are less clear.

Less disruptive substitutions at the X and Y positions might provide important insights into pathophysiology of missense mutations. In the last few years, X- and Y-position substitutions have been identified in individuals with OI [17–21], osteopenia [22], arterial rupture [23,24], classical Ehlers-Danlos syndrome (EDS) [24,25], and Caffey disease [26]. Often these mutations result in substitutions of cysteine for arginine, but other alterations also occur, and the molecular pathophysiology of non-glycine substitutions in the triple helix remains uncertain. Since cysteine for arginine substitutions are quite common, a particularly puzzling question is whether it is the loss of the charged arginine side chain or the acquisition of the sulfhydryl cysteine side chain (or both) that cause these phenotypic changes.

In the current work we describe an individual with a mild form of OI, who has a unique mutation in one *COL1A1* allele (c.2873G>T, p.Arg958Leu). To understand some of the mechanisms by which non-glycine substitutions could result in OI phenotypes, we compared the effects of this substitution with cysteine substitution for the same arginine (c.2873G>T, p.Arg958Leu) and with two substitutions for glycine residues located in the same region of the triple helix (c.2821G>A, p.Gly941Ser and c.2830G>T, p.Gly944Cys). We refer to these substitutions mostly as Arg780Leu (R780L), Arg780Cys (R780C), Gly763Ser (G763S), and Gly766Cys (G766C), respectively. This “legacy” designation based on the triple helical “address” facilitates direct comparison with substitutions in *COL1A2* and in the triple helical domains of other fibrillar collagens.

In fibroblasts we found that both non-glycine substitutions mimicked the effects of substitutions for glycine in that triple helix folding was delayed, procollagen was retained in the ER and resulted in ER dilation and deficient matrix deposition. These observations suggest that substitutions of at least some Y-position arginine residues can destabilize the triple helix and disrupt procollagen folding in osteoblast ER like glycine substitutions, thereby causing bone pathology.

## Materials and methods

### Human subjects

Human subject sample collection and analysis at University of Washington (UW) was approved by the Human Subjects Institutional Review Board (IRB), UW. Clinical samples were transferred to a UW research repository with a waiver of consent for participation in research studies by the UW IRB. Analysis of anonymized samples from the UW repository at NIH was approved by the NIH Office of Human Subject Research.

### Collagen purification and analysis

Dermal fibroblasts were grown from punch biopsies obtained with appropriate consent. Normal control cells from multiple individuals of different ages were used to identify the normal range of variation in different experimental observations [27]. Normal controls shown in the figures were mostly based on CRL-2127 cells purchased from American Type Culture Collection (Manassas, VA). Fibroblasts were grown to confluence at 37 °C in Dulbecco’s modified Eagle’s medium containing 10% fetal bovine serum and 2 mM Glutamax (Invitrogen) in the presence of 5% CO_2_. The cells were incubated for 24 hours in DMEM/Glutamax with 0.1% fetal bovine serum and 250 μM of 2-Phospho-L-ascorbic acid trisodium salt (Fluka). The harvested medium was buffered with 100 mmol/L of Tris-HCl (pH 7.4), protected with protease inhibitors, and used for purification of collagen [27]. Pepsin-digested secreted, intracellular and matrix collagens were prepared [27].

For analysis of overmodification, collagen and procollagen were metabolically labeled with [^3^H]proline (GE Healthcare) and analyzed by SDS-polyacrylamide gel electrophoresis (SDS-PAGE) [28]. For analysis of CNBr peptides, the chains of type I collagen were separated under non-reducing condition in 5% SDS-PAGE gels, the regions of the bands were excised from the gel, treated with CNBr mixture and then set atop a second 12.5% SDS-PAGE gel and analyzed [28].

Amino acid analysis to quantify lysine and hydroxylysine was contracted with Alphalyse protein analysis service (Palo Alto, CA), which performed the analysis by high pressure liquid chromatography.

To demonstrate collagen posttranslational overmodification, pepsin-purified type I collagen from the medium of either control cells or cells from the patient was fluorescently labeled either by AlexaFluor 488 (AF488, Invitrogen) or Cy5 (GE Healthcare) [29]. Then the mixtures of NC_AF488/NC_Cy5, NC_AF488/mutant_Cy5 and NC_Cy5/mutant_AF488 were analyzed after separation of chains by SDS-PAGE.

Kinetics of collagen triple helix folding and clearance from fibroblasts were measured by pulse metabolic labeling with azidohomoalanine in methionine-free media followed by chase in media with 10 mM of methionine [30].

Collagen thermal stability was determined by sensitivity to proteases [31] and by differential scanning calorimetry (DSC) at 0.125 °C/min heating rate. [32]. Procollagen was examined in phosphate buffered saline (PBS), pH 7.4. Pepsin-purified collagen was examined in 0.5 M glycerol, 0.2 M Na-phosphate, pH 7.4 (PGB) to inhibit collagen aggregation and fibrillogenesis [32]. DSC thermograms of collagen in PBS were corrected for the contribution of type III collagen and corrected by -1.7 °C to represent collagen stability in PBS as described previously [27,32]. Denaturation thermogram of pepsin-purified intracellular α1(I)-G763S collagen in PGB was measured by differential scanning circular dichroism [27], demonstrating similar content of mutant and normal molecules in intracellular and secreted collagen. Local reversible unfolding was studied by circular dichroism [27].

### Electron microscopy (EM) and Raman micro-spectroscopy

Cells were cultured for 3 weeks on an ACLAR^®^ fluoropolymer film in DMEM that containied 10% FBS and 250 μM 2-Phospho-L-ascorbic acid trisodium salt, media was changed every 2–3 days. For EM, matrix was fixed with 2.5% glutaraldehyde in 130 mM sodium cacodylate buffer, postfixed in 2% OsO_4_, processed into Spurr's epoxy, sectioned, stained with UO_2_-acetate and Pb-citrate, and examined in a JEOL 1400 electron microscope at NICHD Imaging core. For Raman spectroscopy, cell matrix was fixed with 0.7% formaldehyde solution in 0.7x PBS. Relative ratios of matrix collagen to cell organics were determined from Raman spectra within at least 10 different matrix regions as described in [33].

### Collagen cleavage with soluble MMPs

We studied cleavage kinetics of the mutant and normal control collagens by recombinant human MMP1 (gift of Prof. H. Nagase, Imperial College, London), the catalytic domain of MMP1 (Enzo Life Sciences), and MMP2 (EMD Biosciences). MMP1 was activated with 1 mM 4-aminophenylmercuric acetate [34]. For these experiments, purified type I collagen from the medium of either control cells or cells from the patient was fluorescently labeled either by AlexaFluor 488 (AF488, Invitrogen) or Cy5 (GE Healthcare) [29]. Binary mixtures of mutant-AF488/control-Cy5 and mutant-Cy5/control-AF488 in 50 mM Tris, 150 mM NaCl, 10 mM CaCl_2_, 0.05% Brij were prepared and co-processed with the enzyme at different temperatures. The cleavage products and kinetics were examined on precast 3–8% Tris-Acetate gels (Invitrogen) using the FLA5000 scanner (Fuji Medical Systems) for fluorescence detection and ScienceLab software supplied with the scanner for quantitative analysis.

### DNA sequencing

Genomic DNA and RNA and cDNA were prepared by standard methods. Primers and conditions for amplification of genomic and cDNA and for automated sequencing are available upon request.

### Quantitative real time PCR (qPCR)

RNA was isolated from cells using Purelink RNA kit (Life Technologies), reverse transcribed with SuperScript III First Strand Synthesis Supermix and random hexamers as primers (Invitrogen), and analyzed in a 7500 Fast Real Time PCR system (Applied Biosystems) with Taqman gene expression assays. The same C_T_ threshold value was used for all samples. Relative mRNA quantity was calculated from ΔΔC_T_ values assuming 100% PCR efficiency [35].

### Western blotting

Cells were grown to confluence and incubated with fresh medium that contained 250 μM ascorbate one day before collection. At collection, cells were rinsed with PBS and lysed in 2% sodium dodecyl sulfate, lithium dodecyl sulfate, 50mM dithiothreitol, 50mM Tris, 150mM NaCl, 5mM EDTA, 1mM phenylmethylsulfonyl fluoride, 5mM benzamidine, 10mM *N*-ethylmaleimide, and PhosStop (Roche). Samples were denatured at 95°C for 10 minutes, loaded onto 3–8% Tris Acetate or 12% Bis-Tris gel and transferred onto a 0.45 um nitrocellulose membrane. The blots (Figures A-C in S1 File) were labeled with primary antibodies against proα1(I) C-propeptide (LF-42, a gift of Dr. Larry Fisher, NIDCR, NIH), BIP (Cell Signaling, 3177), PDI (Enzo Life Sciences, ADI-SPA-891-D), Calnexin (Abcam, ab10286), Calreticulin (Cell Signaling, 12238), EIF2α (Cell Signaling, 2103), phosphorylated EIF2α (pEIF2α, Cell Signaling, 3398 and 9721), and b-actin (Abcam, ab8224). After staining with secondary antibodies conjugated to AlexaFluor dyes (Life Sciences), images were captured in an FLA5000 fluorescence scanner (Fuji Medical Systems, Stamford, CT) and analyzed with Multigauge 3.0 software supplied with the scanner.

### Molecular modeling

The structural models were based on Protein Data Bank entry 1BKV [36] and molecular modeling and image generation used UCSF Chimera [37]. Leucine substitution was modeled based on the Dunbrack backbone-dependent library [38].

## Results

### Clinical summary and mutation identification

**Fig 1:**
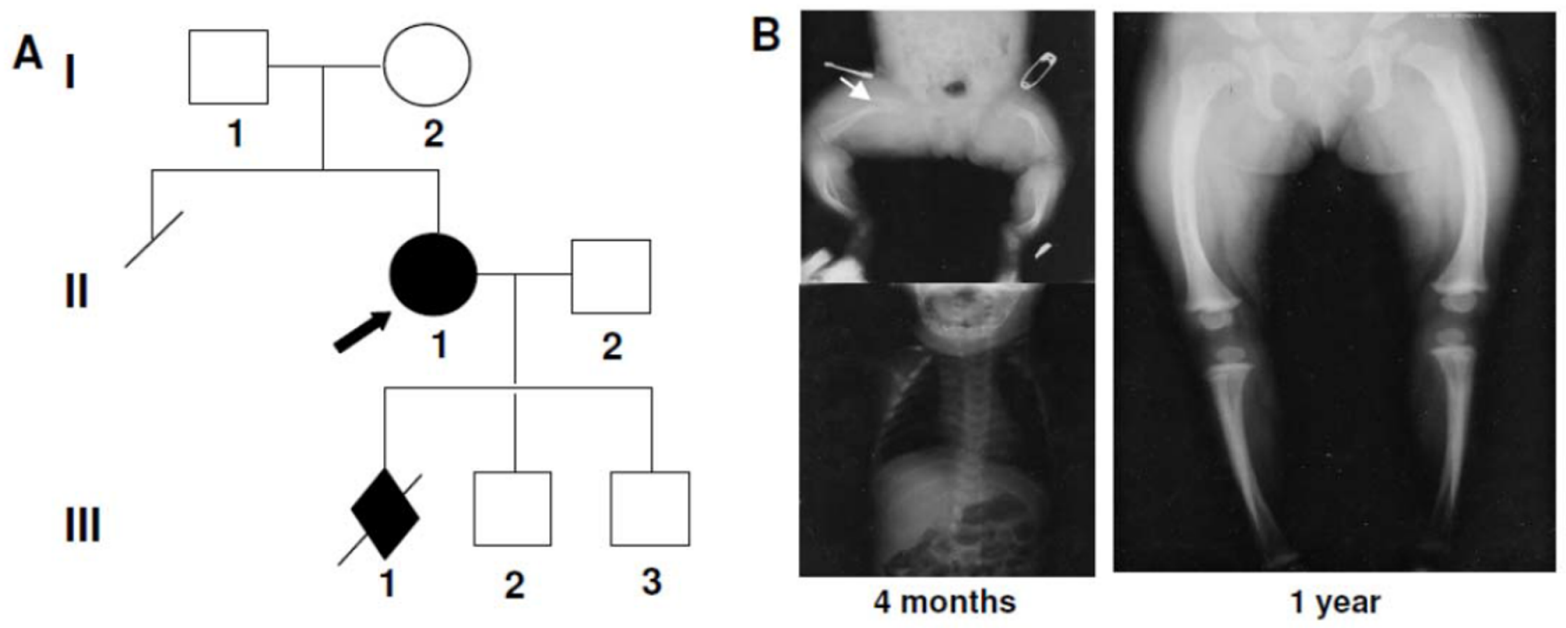
Clinical summary for α1(I)-R780L. A, Family pedigree; the proband (II-1) is marked with arrow. B, radiographs of the proband at 4 months (left) and 1 year (right) of age.

**Fig 2:**
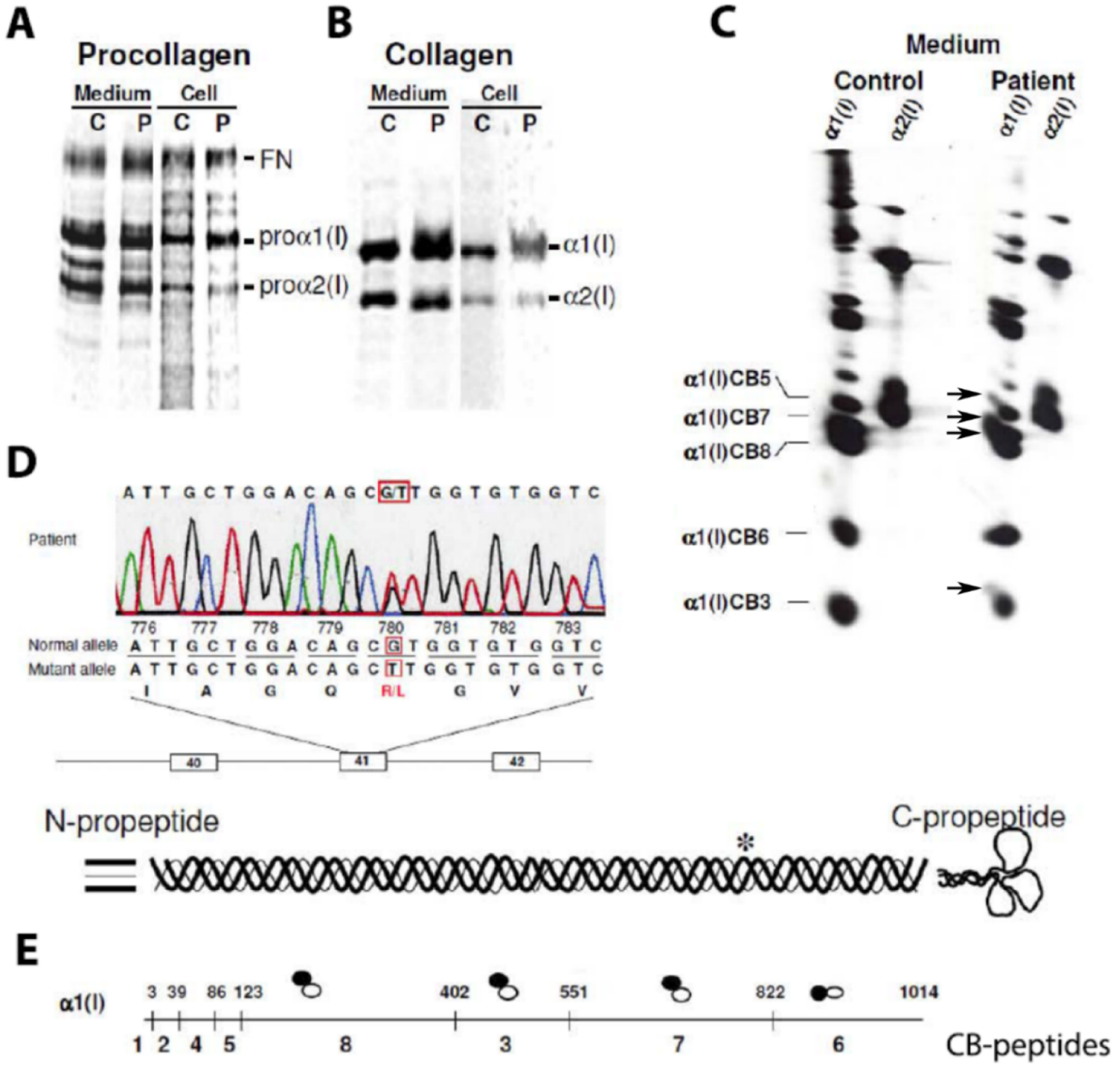
Analysis of procollagen (A) and collagen (B) molecules synthesized by the patient (P) and control (C) cells and mutation identification (C-E). A,B, SDS-PAGE run with 2 M urea to enhance the difference in migration between overmodified and normal chains. Compared to control cells, proα1(I) and α1(I) chains from patient were more heterogeneous and some of the chains had delayed electrophoretic migration due to posttranslational overmodification. C, The shapes of electrophoretic bands of CNBr (CB) peptides from normal (large solid bands) and overmodified (upward streaks marked by arrows) collagen chains. The main bands might be slanted (e.g. control α1(I)) due to imperfect shape/orientation of the band cut out from the original collagen gel for CNBr digestion. The streaks of overmodified chains in the top left corner of the main bands are distinguished by their different orientation. The presence of overmodified α1(I)CB3, α1(I)CB7 and α1(I)CB8, but not α1(I)CB6, is consistent with a defect in folding within residues 551–821 of the triple helical domain (see panel E), that is, amino-terminal to the beginning of α1(I)CB6. D, The heterozygous mutation, c.2873G>T in exon 41 of one *COL1A1* allele, resulted in substitution of the arginine by leucine at position 958 of the protein (p.Arg958Leu), which is position 780 of the triple helical domain (Arg780Leu). E, The figure of CB-peptides shows the location of the methionyl residues in the α1(I) chain (vertical bars). The open circles (○) represent the electrophoretic mobility of the peptides derived from the normal chains relative to the streaks of overmodified peptides from the mutant chains (closed circles, ●).

The index patient (Fig 1A, II-1) was recognized at birth to have a mild form of OI. Neither of her parents was affected with OI (Fig 1A, I-1, I-2). Parental DNA was not available for mutation analysis. At 4 months of age, the proband had an acute fracture of the right femur (Fig 1B, arrow). Radiographic studies at the time demonstrated that the femurs and the tibias were short and bowed; the ribs had near normal appearance although the chest was slightly small. Within a year, the femurs had grown, the bowing was diminished, and the cortex was well mineralized (Fig 1B). As an adult, she had blue sclerae, modest number of fractures, and some bowing of the tibias and is at the 25th percentile for height. Her first pregnancy (Fig 1A, III-1) was terminated at 19 weeks gestation because of the identification of an encephalocele and bowed bones by ultrasound examination. This fetus was heterozygous for the maternal mutation. The encephalocele was considered to have a different basis. Later, her second and third pregnancies were normal by ultrasound examination. Cultured CVS cells from each fetus synthesized only normal type I procollagen and analysis of DNA confirmed that neither carried the mutant allele (Fig 1A, III-2 and III-3). Healthy infants were born subsequently.

Cultured dermal fibroblasts from the proband (II-1) and the first pregnancy (III-1) but not the second pregnancy (III-2) produced both normal and abnormal type I collagen molecules with chains that had slow mobilities (Fig 2A and 2B). The shift in electrophoretic mobility was blocked by addition of α,α-dipyridyl (data not shown), which interferes with the post-translational hydroxylation of lysyl and prolyl residues in the triple helical domain. Analysis of the cyanogen bromide peptides derived from the chains showed that in the α1(I) chains overmodification began in peptide α1(I)CB7, which comprises position 551–821 of the triple helical domain, indicating that an alteration in that domain delays helix propagation (Fig 2C and 2E).

Sequence of the full length of the genomic DNA from *COL1A1* and *COL1A2* revealed a unique mutation in one allele of *COL1A1*: c.2873G>T, p.Arg958Leu, Arg780Leu in the triple helical domain (Fig 2D). The same mutation was identified in cells from the affected fetus (III-1) but was absent in III-2 and III-3 (data not shown). This variant has not been reported in any major population databases (evs.gs.washington.edu/EVS; gnomad.broadinstitute.org/) or ClinVar (https://www.ncbi.nlm.nih.gov/clinvar). No other coding sequencing or splice site mutations were found in either type I collagen gene and no alterations in mRNA sequence or structure of those genes was identified. The asymmetric overmodification is consistent with an altered sequence in the collagens and not in the modifying enzymes. We have used the triple helical or legacy “address” (e.g., residue 780) to identify mutation positions throughout the paper.

The *COL1A1* c.2872G>T, p.Arg958Cys (Arg780Cys) mutation was identified in 1994/95 in a 7 wk old patient with bilateral subdural hematomas and multiple fractures, including vertebral compression fractures. The patient had osteopenia, normal sclerae, and no evidence of Wormian bones or other bone malformations. SDS-PAGE did not show significant posttranslational overmodification of type I collagen; but it revealed evidence of a disulfide bond between α1(I) chains, which was mapped to α1(I)CB7 CNBr peptide. Because of child abuse concern (mother admitted to some abusive behavior) and lack of other individuals with the same mutation, the cause of the fractures remained uncertain. There was no patient follow up. Parental DNA was not analyzed.

### Effects of α1(I)-R780 substitutions on procollagen and collagen thermal stability

Substitutions of obligatory glycine residues decrease the thermal stability of the whole molecule and lower its apparent denaturation temperature (T_m_) from ~0.5 °C up to 5 °C [27]. Except for the immediate vicinity of the N- and C-terminal ends of the triple helix, those substitutions reduce the thermal stability of procollagen to exactly the same extent as collagen. Several examined substitutions for Y-position Arg residues were also reported to decrease collagen T_m_, all by approximately 1 °C [17,23].

**Fig 3:**
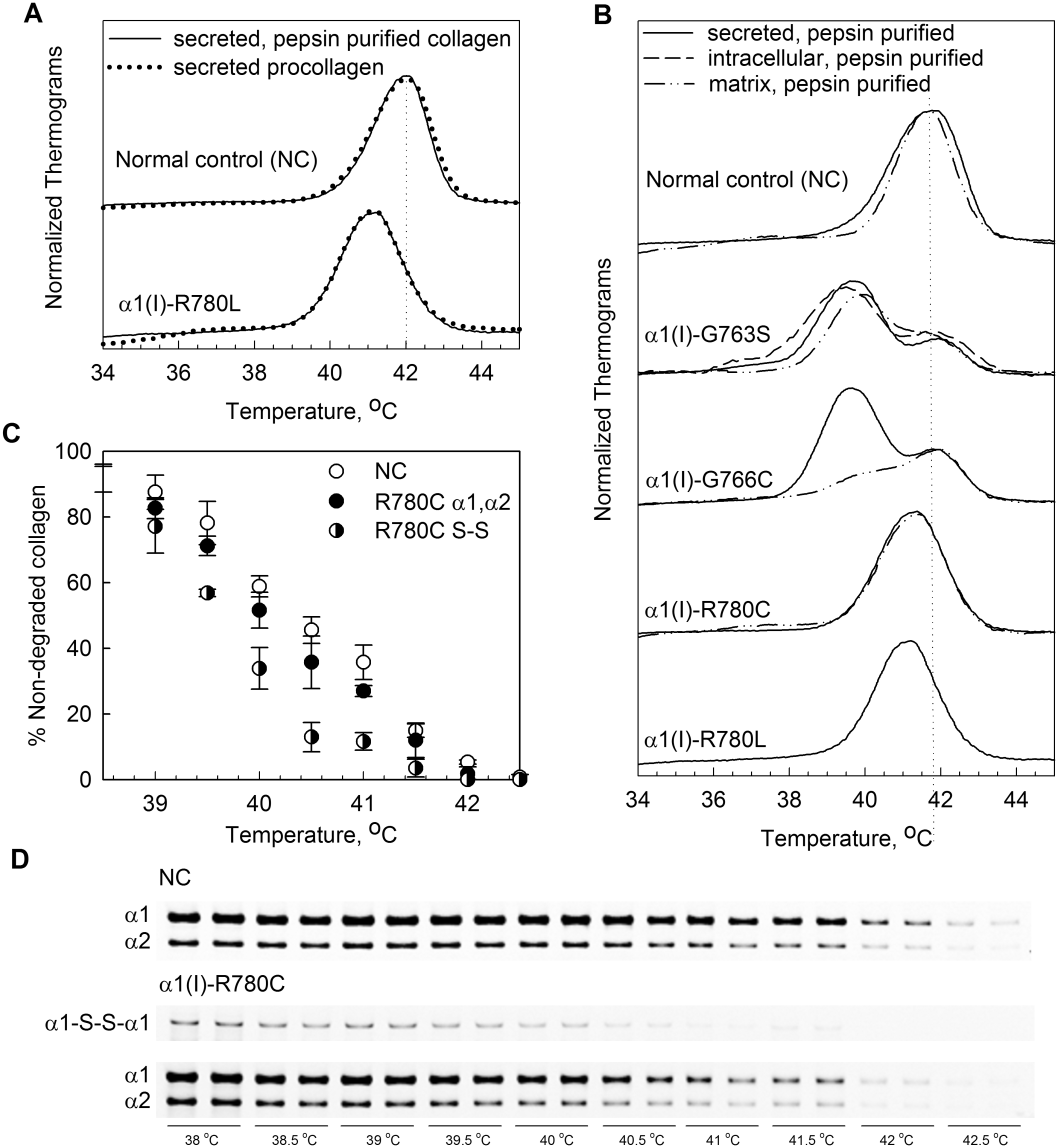
Type I collagen triple helix destabilization caused by glycine and arginine mutations. A, DSC denaturation thermograms of pepsin-purified collagen (solid lines) and procollagen (dotted lines) secreted into media by normal control (NC) and patient cells. B, Denaturation thermograms of pepsin-purified collagen from media (secreted, solid lines), cell layer (intracellular, dashed line), and extracellular matrix deposited by cells (matrix, dash-dotted lines). C,D, Analysis of α1(I)-R780C collagen thermal stability by enzymatic digestion and gel electrophoresis. In A and B, each thermogram peak represents denaturation of molecules with or without mutant chains. The peak maximum is the corresponding apparent denaturation temperature T_m_, which is the same in collagen and procollagen molecules (for NC as well as most mutants) [27]. A heterozygous Gly substitution in the α1(I) chain might result in up to 3 peaks on the denaturation thermogram, representing molecules with 2, 1, and 0 mutant chains. In α1(I)-G766C collagen, the molecules with 1 and 2 mutant chains have the same T_m_, producing one peak at ≈39.5 °C; the molecules without mutant chains produce the second peak at ≈42 °C. The same is true for α1(I)-G763S collagen. In α1(I)-R780L or α1(I)-R780C, molecules with 1 and 2 mutant chains produce a peak at ≈41 °C; the 42 °C peak of molecules without the mutant chains is too close to 41 °C, so that it is not resolved. The area under each peak is proportional to the fraction of the corresponding molecules. A change in this fraction upon secretion from cells or incorporation into matrix alters the shape of the denaturation thermogram [27]. A reduced intensity of the 39.5 °C peak in matrix vs. secreted collagen indicated that only 25% of α1(I)-G766C molecules in extracellular matrix contained mutant chains, but no effects of other mutations on collagen secretion or matrix incorporation were detected. In C and D, thermal stability of secreted collagen was measured by 2 min equilibration at different temperatures followed by 5 min at room temperature, 1 min digestion with trypsin/chymotrypsin mixture, and separation of chains by gel electrophoresis at non-reducing conditions as described in [31]. Panel C shows quantitative analysis of gel electrophoresis (D), each point representing an average of 4 experiments.

Analysis of secreted collagen and procollagen by differential scanning calorimetry (DSC) and by sensitivity to trypsin and chymotrypsin revealed 0.5–1.0 °C lower T_m_ in α1(I)-R780L than in the control (Fig 3). DSC denaturation thermograms showed that the effects of α1(I)-R780L and α1(I)-R780C substitutions on the thermal stability of collagen were identical. The molecules with a Cys780-S-S-Cys780 disulfide bond appeared to have ~0.5 °C lower stability than the molecules without this bond (Fig 3D).

Substitutions for glycine residues α1(I)-G763S and α1(I)-G766C lowered collagen T_m_ by 2–2.5 °C. The effect of substitutions for glycine residues on the collagen T_m_ is determined by their location along the triple helix rather than the identity of the substituting residue [27]. Comparison of the α1(I)-R780L and α1(I)-R780C collagens suggested that the same might be true for substitutions that do not alter triple helical glycine residues.

### Local destabilization of the triple helix at the MMP cleavage site

Measurement of collagen T_m_ by either DSC or chymotrypsin-trypsin digestion shows only mutation effects on the stability of the whole molecule. To investigate the extent of local destabilization of the triple helix near the mutation site, we exploited the proximity of the mutation to the collagenase MMP1 site (residues 775–776 of the triple helix) and the requirement of triple helix unwinding prior to cleavage by the enzyme.

**Fig 4:**
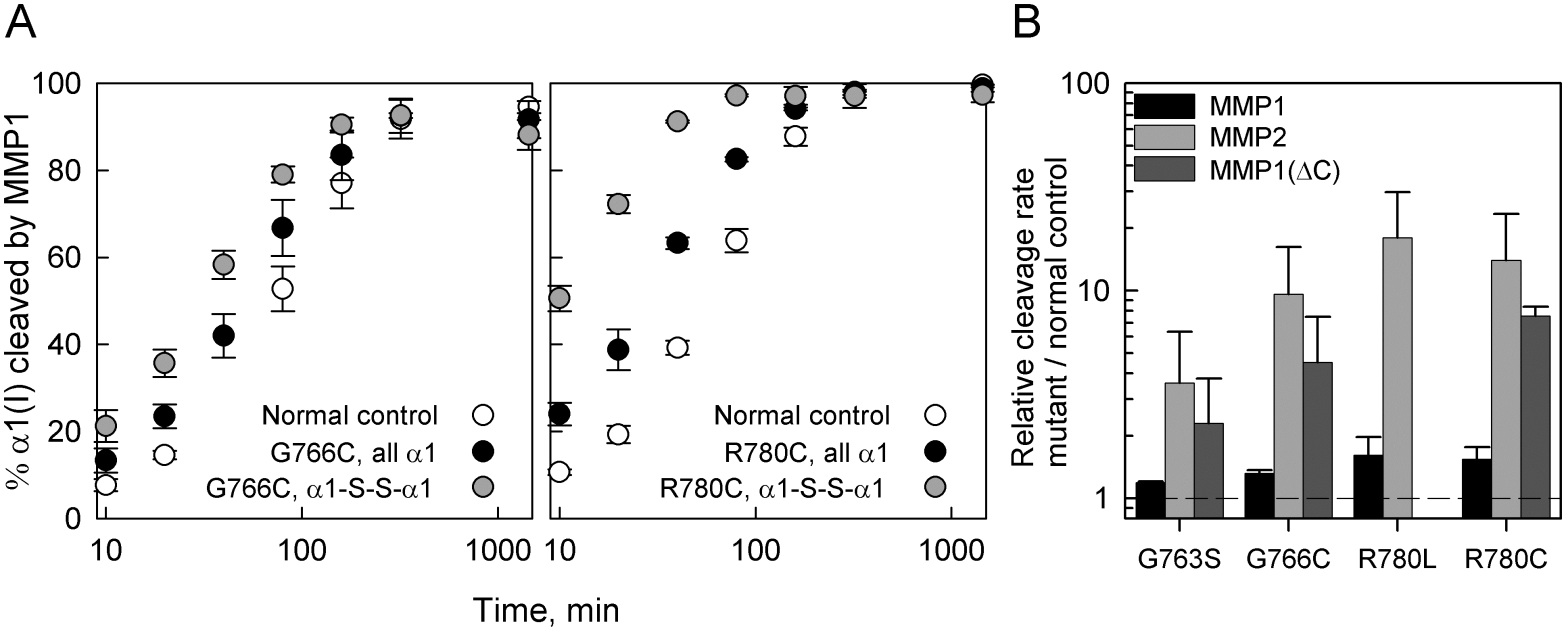
Cleavage of mutant collagens by MMP1, MMP2, and catalytic domain of MMP1 (MMP1(ΔC)). A, cleavage kinetics of collagens by MMP1 in molecules with Cys for α1(I) Gly or Cys for α1(I) Arg substitutions. Each time point represents an average of two experiments. In one, the mutant labeled with Alexa Fluor 488 (AF488) was mixed with normal control labelled with Cy5 and treated with MMP1 within the same sample tube. In the other, the fluorescent labels were switched. The error bar represents the corresponding standard deviation. Gray circles show the kinetics of cleavage for disulfide bonded chains formed in molecules containing two α1(I) chains with Cys for Gly or Cys for Arg substitutions. B, relative cleavage rates for all studied mutants determined as the ratio of the initial cleavage rate of all α1(I) chains in the mutant to the initial cleavage rate of α1(I) chains in the normal control within the same sample tube. The initial cleavage rates were determined by linear regression of the first 3–4 data points in the kinetic experiments shown in A and similar experiments for the other mutants and enzymes. The cleavage rates of α1(I)-R780L with MMP1(ΔC) were not measured. The cleavage rates of all mutants were larger than in normal control with high statistical significance (*p*<0.001). For instance, the cleavage rate by MMP2 was 0.18±0.01 in α1(I)-R780L vs. 0.01±0.006 in NC (in the same units), yielding high statistical significance for (0.18±0.01)/(0.01±0.006) > 1 despite noticeable uncertainty in the absolute value of this ratio.

We found an increase in the cleavage rate of α1(I)-G763S collagen by MMP1 (Fig 4B, black bars). The increase in the cleavage rate was more pronounced for α1(I)-R780L, α1(I)-R780C and α1(I)-G766C collagens. Overall, α1(I)-R780L and α1(I)-R780C substitutions had similar effects, indicating that the global and the local changes in the triple helix stability were similar. In α1(I)-G766C and α1(I)-R780C collagens, the cleavage rate of molecules with the two α1(I) chains linked by a disulfide bond was faster than molecules with either one or no altered chains, consistent with more significant triple helix opening in these molecules (Fig 4A).

We observed similar effects of the mutations on collagen cleavage by gelatinase MMP2 (Fig 4B, light grey bars) and by the catalytic domain of MMP1 (MMP1(ΔC)) (Fig 4B, dark grey bars). In short, the collagens that contained α1(I)-G766C, α1(I)-R780L, or α1(I)-R780C chains were much more susceptible to cleavage than normal control; α1(I)-G763S collagen was less susceptible than other mutations. The quantitatively larger effects of the mutations on collagen cleavage by MMP2 and MMP1(ΔC) confirmed that there was local triple helix destabilization at the cleavage site, since these enzymes are less able to cut the intact triple helix than MMP1 [34]. The susceptibilities of α1(I)-R780L and α1(I)-R780C collagens to cleavage were similar, consistent with similar local destabilization of the triple helix in addition to similar change in T_m_ for the whole molecule.

**Fig 5:**
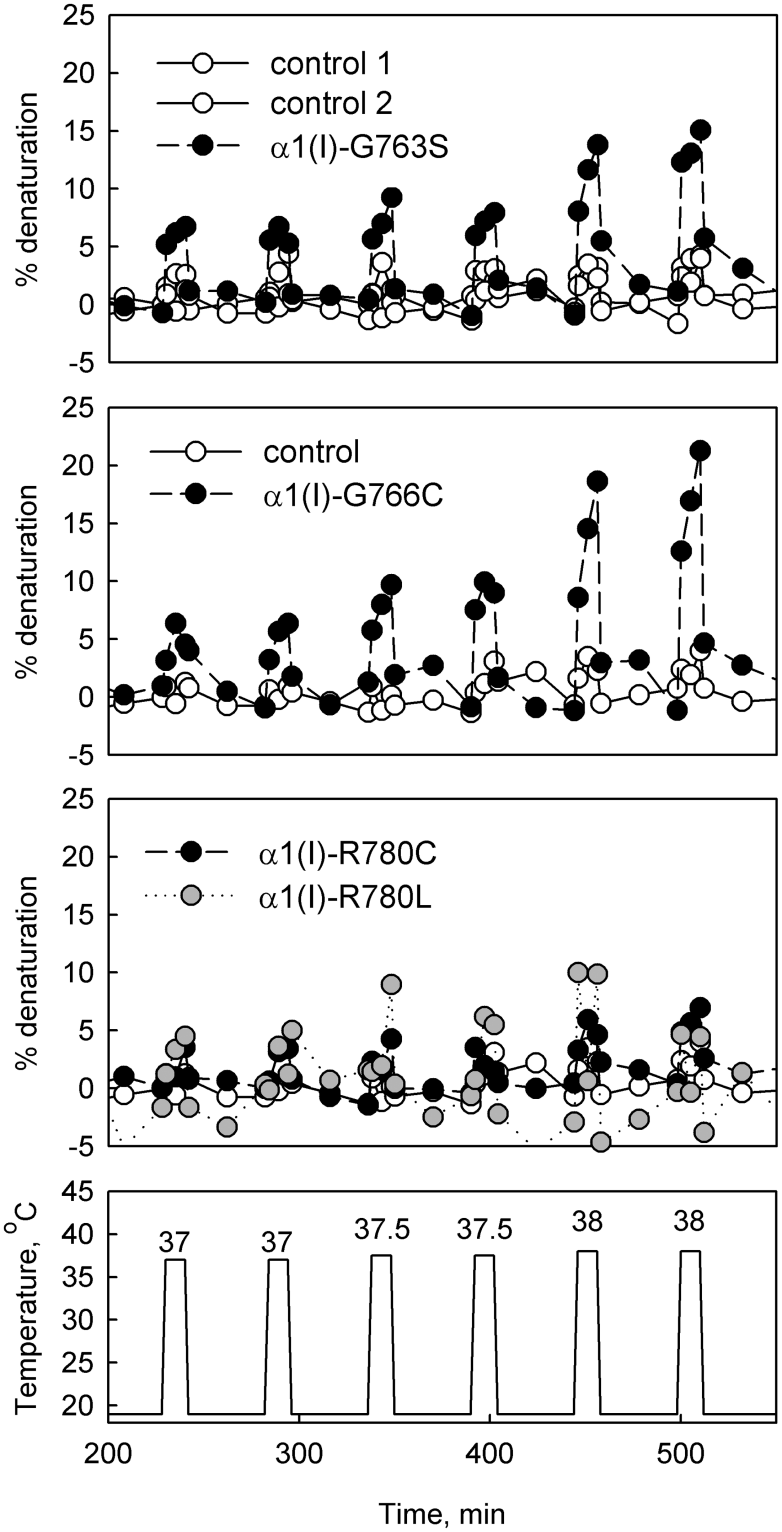
Reversible unfolding of mutant collagens. The fraction (% denaturation) of the triple helix length unfolded in response to a train of heating pulses (bottom figure). This fraction was measured by monitoring % change in ellipticity of collagen solution in 2 mM HCl (pH 2.7) at 223.8 nm compared with the ellipticity change upon complete denaturation. Complete denaturation of collagen triple helix under the same conditions (e.g. after a 55 °C heating pulse) was irreversible. Reversible changes in the ellipticity were therefore associated with unfolding of a region(s) within the helix rather than the whole molecule. Assuming ellipticity of the unfolded region being the same as of a fully unfolded collagen chain and the ellipticity of the folded region being the same as of a fully folded triple helix, the measured % change in ellipticity represents the % of the triple helix length unfolded in response to the heating pulse. Thus, ~10% reversible change in the ellipticity observed in α1(I)-G763S and α1(I)-G766C collagen solutions indicated unfolding of 10% of the triple helix length.

To further understand the extent of the local helix destabilization, we measured circular dichroism (CD) spectra of purified collagen in response to trains of heating pulses, during which the temperature was clamped at 37, 37.5, and 38 °C (Fig 5). At these temperatures there is rapid unfolding of unstable triple helical regions, which is reversible upon cooling, provided that the pulse is short enough to prevent denaturation of the entire molecule. The relative change in the spectra at 223.8 nm during the heating pulse is proportional to the unfolded fraction of the triple helix [27,29].

Reversible unfolding of up to 10% of the triple helix (~100 amino acids) was observed for α1(I)-G763S and α1(I)-G766C collagens (Fig 5). In contrast, CD spectra of collagens with α1(I)-R780 substitutions were similar to the normal control, placing an upper limit of ~ 3% (~ 30 amino acids) on the length of the region destabilized by the mutations. Compared to α1(I)-G763S and α1(I)-G766C, the triple helix disruptions produced by α1(I)-R780 substitutions were just as large or larger at the adjacent MMP cleavage site. However, these disruptions did not propagate as far along the triple helix, probably because of highly heterogenous structural stability of different regions within collagen molecule [27].

### Procollagen folding delay

Local destabilization also means that formation of the triple helix within this region during procollagen folding becomes unfavorable. The triple helix folds in a zipper-like fashion from the C- to N- terminal end, and local instability causes a pause around the mutation site until the helix is renucleated beyond this region [39]. This folding delay leads to asymmetric posttranslational overmodification, N-terminal to the mutation. The overmodification of CNBr peptides from α1(I) chain regions N-terminal to the mutation was consistent with such a folding delay in α1(I)-R780L procollagen (Fig 2).

**Fig 6:**
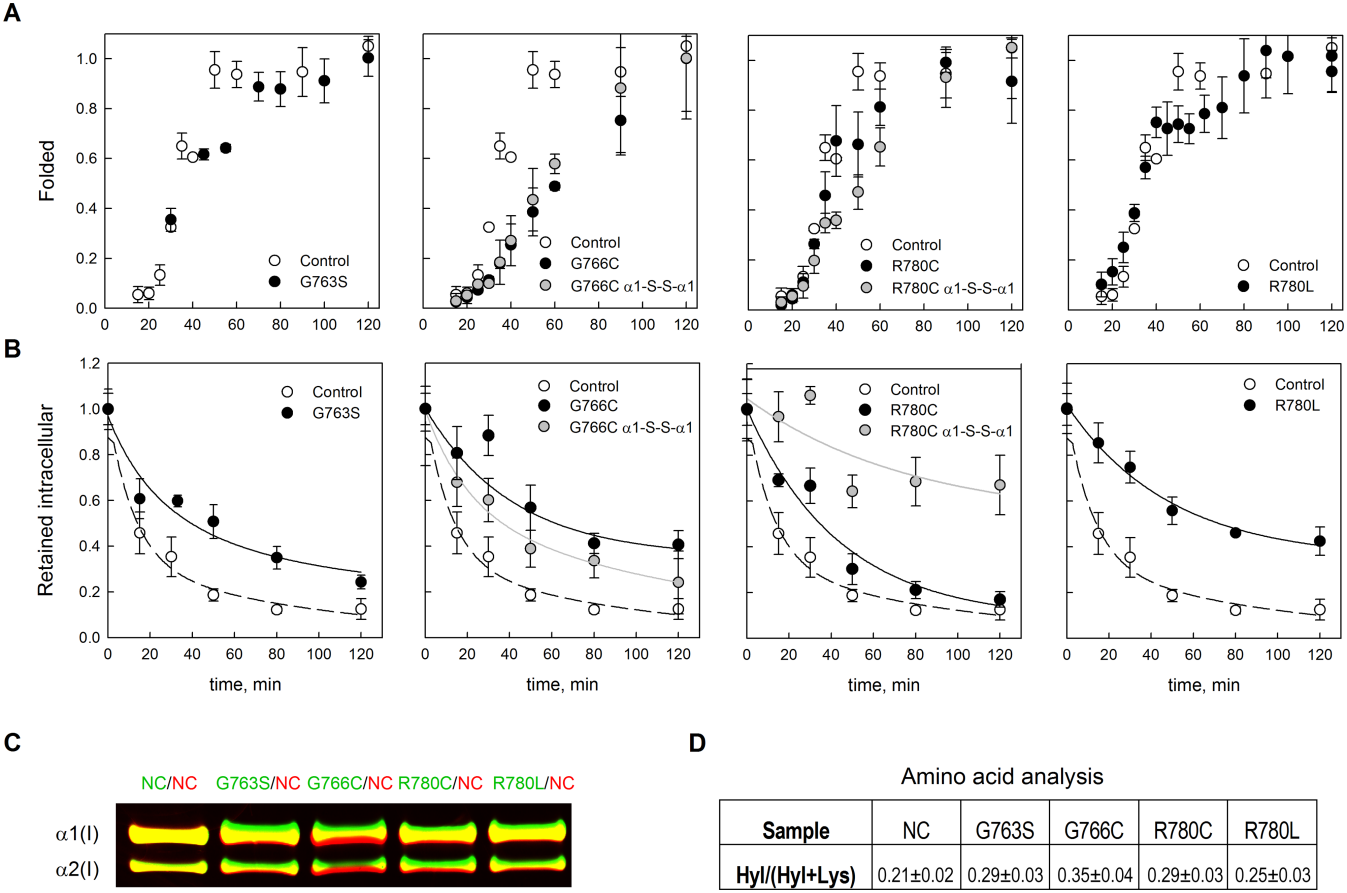
Delay in type I collagen folding (A) and intracellular retention (B) caused by substitutions for glycine and arginine in the triple helical domain of proα1(I) chains. A, Collagen triple helix folding measured by incorporation of azidohomoalanine (Aha) into newly synthetized collagen chains after 10 min Aha pulse, followed by chymotrypsin-trypsin digestion, labeling with DIBO dye, and quantifying α1(I) chains resistant to chymotrypsin-trypsin digestions by SDS-PAGE. The fraction of folded (chymotrypsin-trypsin resistant) molecules was measured from the intensity of Aha-DIBO-labeled α1(I) bands on SDS-PAGE relative to the intensity of these bands after 2 h chase. B, Residence time of intracellular Aha-DIBO-labeled collagen after 2 h Aha-pulse. C, Visualization of a shift in migration of overmodified mutant molecules vs. normal (NC) molecules on SDS-PAGE by double fluorescent labeling. Binary mixtures of AlexaFluor488-labeled NC, G763S, G766C, R780L, or R780C (green) with Cy5-labeld NC (red) were used to compare the migration of mutant vs normal control in the same SDS-PAGE lane. Relative to Fig 2B, the shift is less pronounced since urea was not used to enhance the separation, but it is still clearly visualized by the separation of red and green colors. D, Amino acid analysis shows higher fraction of hydroxylysines in all mutant collagens. The standard deviation was measured in NC (5 samples) and assumed to have similar relative value in other collagens.

Using pulse-chase methionine substitution with a synthetic azidohomoalanine (Aha) analog followed by fluorescent labeling of Aha [30], we directly measured procollagen folding rate in human fibroblasts (Fig 6A). We confirmed that in these instances substitutions for arginine and glycine delayed triple helix folding. The most pronounced delay was observed for α1(I)-G766C. Interestingly, α1(I)-G766C molecules with the Cys766-S-S-Cys766 disulfide bond had shorter folding delay than α1(I)-G766C molecules without the bond; α1(I)-R780C molecules with the Cys780-S-S-Cys780 bond had longer folding delay than α1(I)-R780C molecules without the bond (Fig 6A).

The folding data perfectly correlated with posttranslational overmodification that we detected by slower electrophoretic migration on gel using mixtures of mutant and normal control collagens labeled with different fluorophores (Fig 6C) and by increased fraction of triple helical hydroxylysines (Fig 6D). All three parameters increased in the following order NC < R780L < G763S and R780C < G766C.

### Procollagen retention by mutant cells

We then used pulse-chase labeling with Aha to examine the kinetics of procollagen clearance from cells [30]. The half-lifetime of Aha-labeled procollagen inside the cell increased from ~15min in NC to ~45 min in α1(I)-G763S, ~90 min in α1(I)-G766C, ~30 min in α1(I)-R780C and ~75min in α1(I)-R780L (Fig 6B). Consistent with the folding kinetics, the Cys766-S-S-Cys766 bond decreased while the Cys780-S-S-Cys780 bond increased the procollagen clearance time.

**Fig 7:**
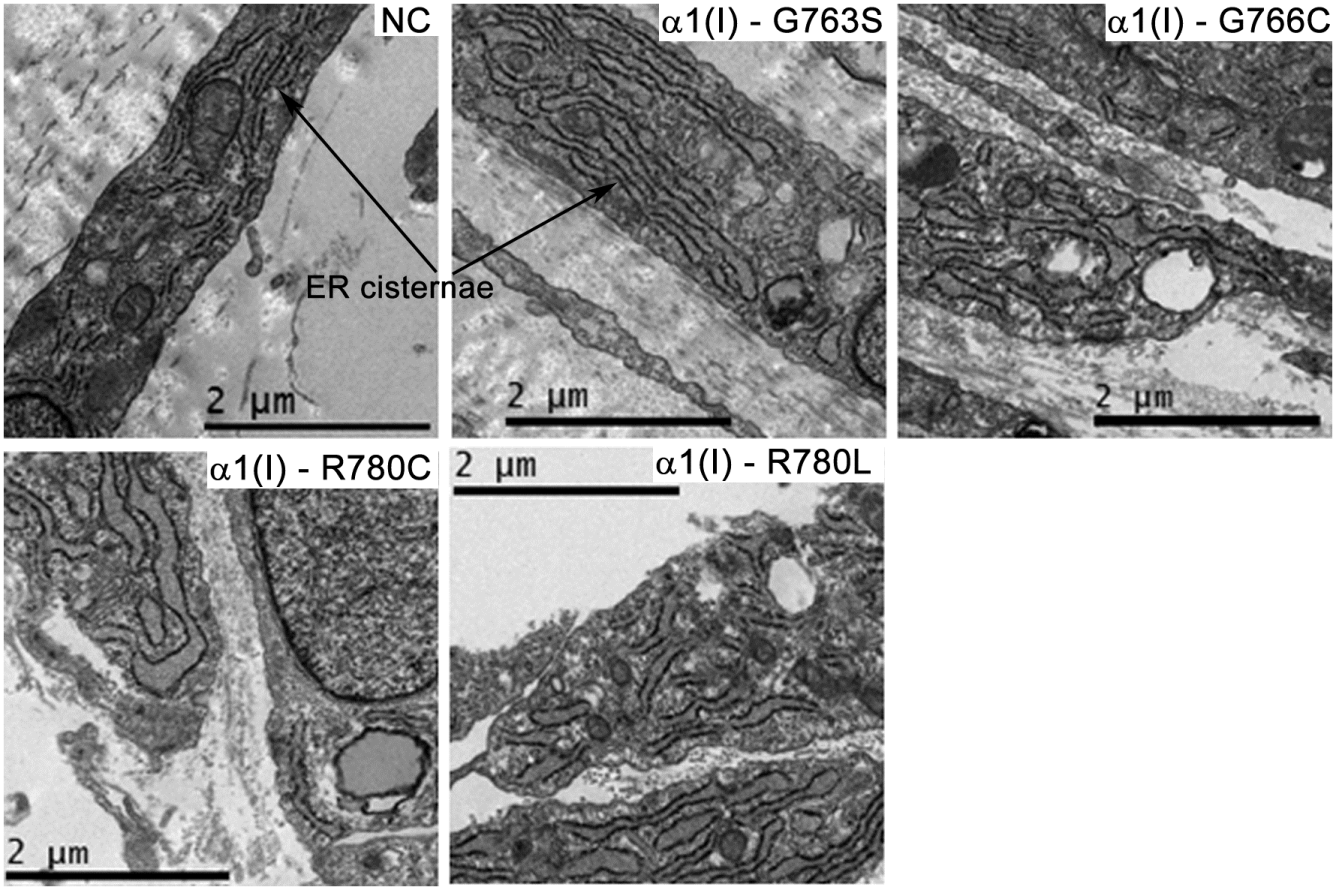
Effect of type I collagen mutations on endoplasmic reticulum (ER) dilation. Representative electron microscopy images of human fibroblasts show dilated ER cisternae in all mutant but not normal control cells. Rough ER cisternae are distinguished by ribosomes (black spots) lining the ER membrane. ER dilation was more subtle in α1(I)-G763S,G766C and more pronounced in α1(I)-R780C,R780L cells.

Larger delays in the clearance time compared to the folding delays indicated retention of misfolded procollagen in the cell. This conclusion was supported by electron microscopy observation of dilated ER cisternae in all mutant but not NC cells (Fig 7). Yet, we found no evidence of significant selective degradation of mutant chains inside the cells. The fractions of mutant chains in secreted procollagen estimated from denaturation thermograms appeared to be consistent with the corresponding expected values and we did not observe significant differences between the thermograms of intracellular and secreted molecules (Fig 3A and 3B).

### Cell stress response

**Fig 8:**
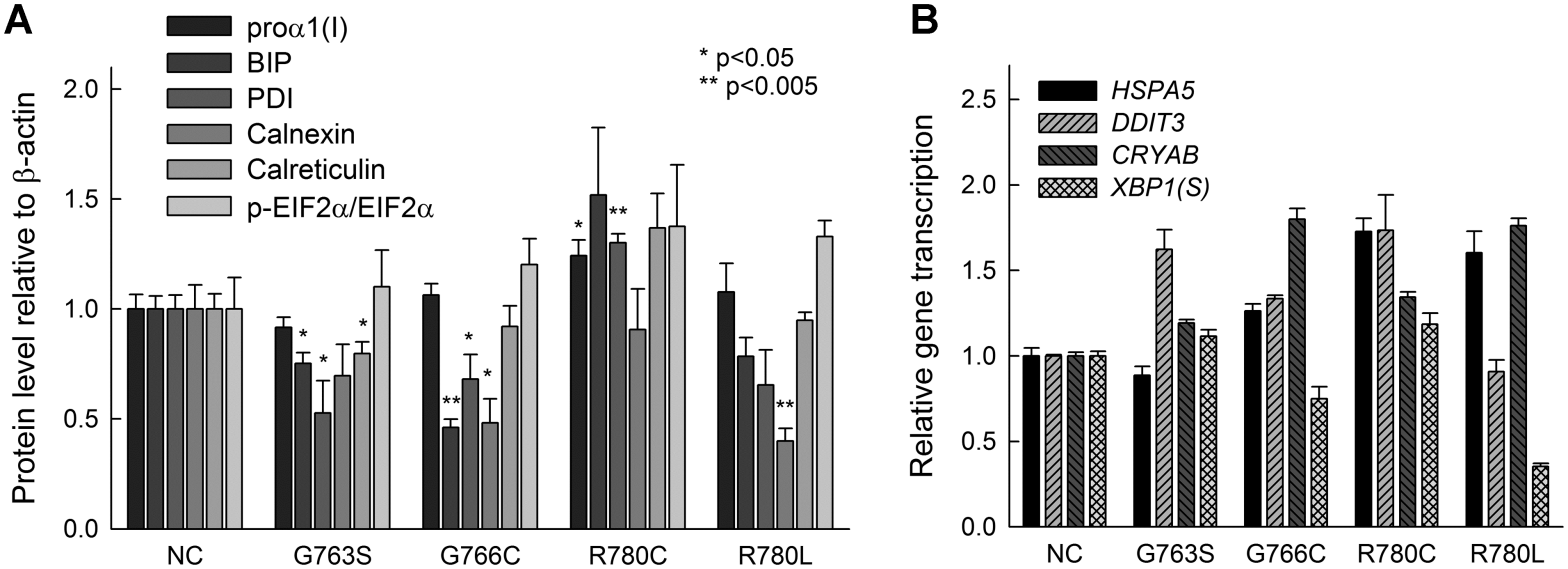
Cellular stress caused by glycine and arginine mutations in type I collagen. A, Markers of integrated cell stress response (p-EIF2α) and unfolded protein response (BIP) as well as ER chaperones PDI, calnexin and calreticulin were measured in biological-triplicate by Western blotting relative to β-actin. B, Unfolded protein response (*HSPA5*/BiP and *XBP1(S)*) and integrated cell stress response (*DDIT3*/CHOP and *CRYAB*/αB-crystallin) mRNA markers were measured in technical-triplicate by qPCR (ΔΔC_T_ method) using a geometric mean of HPRT1, B2M and GAPDH as an endogenous control.

Retention of misfolded procollagen and resulting ER dilation suggested the possibility of cell stress. Western blot analysis (Fig 8A) provided evidence of increased EIF2α phosphorylation expected in cell stress, but this trend did not reach statistical significance. Consistently, real-time quantitative PCR (qPCR, Fig 8B) suggested upregulation of *DDIT3/*CHOP (α1(I)-G763S,G766C,R780C) and *CRYAB*/αB-crystalline (α1(I)-G763C,R780L,R780C). We also observed decreased level of calnexin and PDI in α1(I)-G763S, α1(I)-G766C and α1(I)-R780L cells, indicating some disruption of ER function. Except for α1(I)-R780C cells, we found no evidence of BIP upregulation either by Western blotting or qPCR. We also found no evidence of increased *XBP1* splicing.

### Collagen matrix deposition and structure

**Fig 9:**
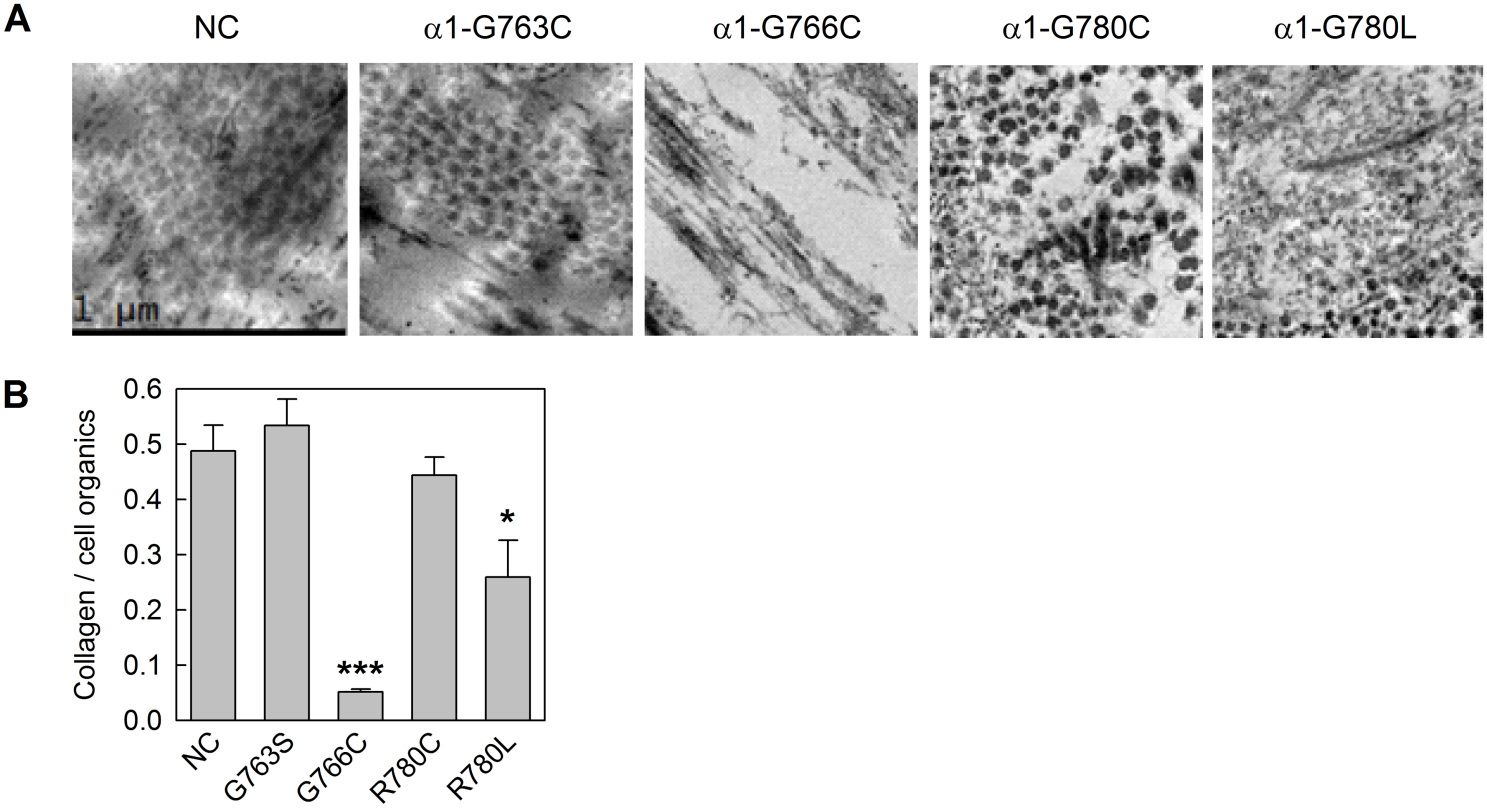
Effect of type I collagen mutations on matrix deposition. A, Electron microscopy images of collagen fibrils deposited during 3-week culture. B, quantitative analysis of collagen content in extracellular matrix relative to cells by Raman microscopy; *, *p*<0.05; ***, *p*<0.001. The collagen/cell organics is the ratio of intensities of collagen-specific Raman bands to cytoplasm-specific Raman bands of organic material, which is present in cells but not in extracellular matrix. This ratio is proportional to the amount of collagen per cell present in the matrix.

Comparison of collagen denaturation thermograms from cell culture media and matrix isolates revealed abnormal matrix incorporation only for α1(I)-G766C molecules (Fig 3B). Consistently, ~10% of the amount incorporated by control cells was deposited into matrix by the cells (Fig 9B) and the resulting matrix contained only very thin, disorganized fibers (Fig 9A). The matrix produced by α1(I)-G763S and α1(I)-R780C cells contained collagen fibers with normal size and appearance as well as normal amount of collagen, although α1(I)-R780C matrix appeared to be less well-organized. The α1(I)-R780L matrix had slightly less collagen and appeared to be more disorganized and heterogenous across the culture than the α1(I)-R780C matrix.

### Molecular model predicts that loss of arginine causes destabilization

**Fig 10:**
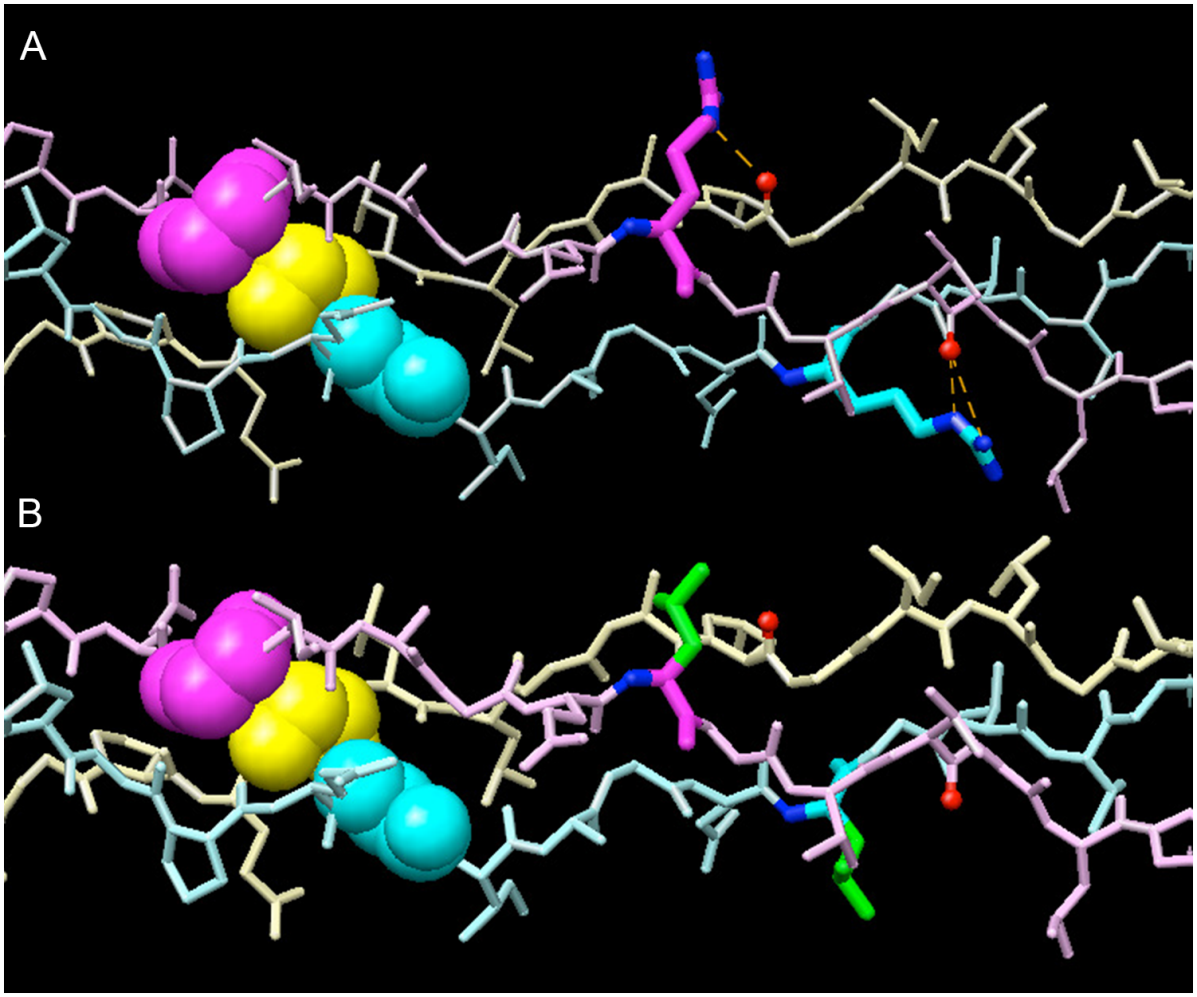
Model of effects of substitution of arginine by leucine at position 780 of the triple helix in the α1(I) chain. A stretch of 14 residues are shown in three chains which in α1(I) are Pro773-Gln-Gly-Ile-Ala-Gly-Gln-Arg-Gly-Val-Val-Gly-Leu-Pro786. The glycines at position 775 are represented by the space filling models in which cyan (chain A) and magenta (chain B) are the α1(I) chains and the α2(I) chain is in yellow (chain C). The chain order is assumed to be α1(I)- α2(I)- α1(I) in the helical domain. A, The normal sequence in which the residue at position 780 of the triple helix is arginine shown in magenta and cyan on their respective chains. The hydrogen bonds are shown by the dotted lines. B, The mutant sequence in which both residues at position 780 are occupied by leucine. No hydrogen bonds form to stabilize the molecule.

To understand how the loss of Arg780 affects the triple helix in the context of the surrounding sequence at this particular site in type I collagen, we performed molecular modeling experiments. Previous studies of homotrimeric model peptides revealed much higher triple helix stability of sequences with Y-position arginine or hydroxyproline compared to other amino acids [40]. Computational analysis [41] and the crystal structure of a triple helical peptide incorporating arginine-containing fragment at the collagenase cleavage site in type III collagen [42] indicated that arginine side chains form hydrogen bonds with backbone carbonyl groups that result in significant stabilization of the triple helix [40]. Our computational analysis of the collagenase cleavage site in type I collagen is most compatible with the presence of hydrogen bonds between the ε-nitrogen of Arg780 on one α1(I) chain and backbone of the α2(I) chain as well as between the ε- and terminal nitrogens of Arg780 on the other α1(I) chain and backbone of the first α1(I) chain (Fig 10A, dashed lines). Leucine or cysteine substitutions for Arg780 (Fig 10B) prevent formation of these hydrogen bonds, destabilizing the helix.

## Discussion

Although *COL1A1* or *COL1A2* mutations that result in substitutions at X or Y-positions in the triple helix are much less common among patients than those that result in substitutions of obligatory glycines, they provide insights into mechanisms by which altered collagens can produce bone pathology in OI. In particular, reduced bone mineral density with or without recurring fractures was observed in most individuals reported with Y position Arg-to-Cys substitutions but not those in which the substitutions were at the X-position. The interpretation of these phenotypic differences has to take into account several considerations. Arg-to-Cys substitutions might lead to bone pathology as a result of osteoblast malfunction caused by misfolding of mutant procollagen molecules and their retention in the ER and/or due to disruption of extracellular matrix properties by incorporation of secreted mutant collagen molecules. Both intra- and extra-cellular effects of the mutation could be related to the loss of a Y-position arginine residue, which stabilizes the collagen triple helix locally [40], serves as a binding site for HSP47 in the process of maturation of the triple helix in the ER [43], and might be important for collagen interactions in the ECM. The gain of a cysteine residue, which is not present in normal triple helices, might lead to detrimental formation of disulfide bonds in the ER, the Golgi, or in the ECM. Finally, the intra- and extracellular effects of free cysteine residues could be different for X- vs. Y-position cysteine residues perhaps because of different preferred conformations of the side chains at these positions and different bond formation efficiencies.

In the family identified here, the α1(I)-R780L substitution occurred at the same site as the α1(I)-R780C substitution in an unrelated individual with mild OI. Although we did not find other patients with the latter substitution, the present study supports its pathogenicity by demonstrating the similarity of the effects of both mutations on collagen properties. Analysis of the physical and chemical properties of collagen secreted by dermal fibroblasts from these patients provided us with a unique opportunity to distinguish the effects of Arg loss from Cys gain. It also enabled us to get additional insights into intra- vs extra-cellular mechanisms of bone pathology in OI.

### α1(I)-R780 is not essential for binding or cleavage of collagen chains by collagenases

One possible effect of α1(I)-R780L or α1(I)-R780C molecules in the ECM could be inhibition of collagen cleavage by collagenases, because Arg780 appeared to be important for interaction of these enzymes with collagen [44]. A recent crystal structure revealed a hydrogen bond between the corresponding Arg residue in the leading chain of a triple helical peptide and MMP1, but substitution of this Arg to Ala did not have a significant effect on the enzyme-peptide interaction [45]. We found that cleavage of α1(I)-R780L and α1(I)-R780C containing molecules was faster rather than slower by MMP1 and MMP2, as a result of triple helix destabilization at the cleavage site. Increased susceptibility to collagenases might affect ECM remodeling, but it is not likely to be responsible for the OI phenotype, since collagenases do not appear to play a major role in bone [46].

### Y-position Arg substitutions destabilize the triple helix and affect procollagen folding

The common feature of OI-causing substitutions of Y-position arginine residues [17,23] is that all of them destabilize and affect the folding of the triple helix, similar to substitutions for glycine. Despite small changes in the collagen T_m_ (Fig 3), the increased susceptibilities to cleavage by MMP2 and catalytic domain of MMP1 (Fig 4) clearly show significant local destabilization of the helix already at 25 °C. This destabilization is comparable to effects of some substitutions for glycine residues described in this and our earlier studies [27]. CD measurements indicate that this destabilization is limited to less than ~ 30 adjacent amino acids (Fig 5). However, it disrupts the propagation of triple helix folding through the mutation site, as indicated by the folding delay (Fig 6A) and posttranslational overmodification of triple helical residues on the N-terminal side of the mutation (Figs 2C & 6C and 6D).

Y-Arg loss is the primary factor in decreasing the triple helix stability while the identity of the substituting amino acid appears to be secondary, as found previously for substitutions of obligatory Gly [27]. Similar effects of Leu and Cys suggest that the destabilization is not associated with detrimental interactions of their side chains. Even formation of a Cys780-S-S-Cys780 disulfide bond has only a relatively minor effect on the helix stability (Fig 3D) and folding (Fig 6C), like the formation of a Cys766-S-S-Cys766 disulfide bond. Instead, studies of triple helical peptides [40], our previous studies [41], and our computer simulations all suggest that the triple helix destabilization is caused by the loss of hydrogen bonds between Y-position Arg side chains and triple helix backbone carbonyls.

Furthermore, the loss of Y-Arg affects procollagen folding not only because it destabilizes the triple helix but also because these Arg residues are essential for HSP47 binding [43], which is required for the final stages of triple helix folding [47–49]. Although α1(I)-R780 is a medium affinity HSP47 binding site, simultaneous triple helix destabilization and loss of HSP47 binding might explain the folding delay and posttranslational overmodification comparable to Gly-X-Y repeat disruption (Figs 2 and 6).

### Y-Arg loss causes ER accumulation of misfolded procollagen similar to Gly substitutions, likely resulting in cell stress and malfunction

Even relatively minor procollagen folding delay (like in α1(I)-G763S, α1(I)-R780C and α1(I)-R780L cells) causes major procollagen misfolding and accumulation of the misfolded molecules inside the cells. Indeed, the half-time required for clearing procollagen molecules from all these cells increases more than two-fold. Although α1(I)-G763S, α1(I)-R780C and α1(I)-R780L mutations cause more subtle changes in the folding rate than α1(I)-G766C, their effects on the clearance kinetics are much more pronounced and similar (Fig 6B). Curiously, neither Y-Arg nor Gly substitutions we studied significantly reduce the fraction of mutant chains in secreted procollagen. The fraction of the molecules retained inside the cells might not be large enough for detecting the change in the chain composition in secreted procollagen from denaturation thermograms. Moreover, mutant cells might non-selectively retain molecules with and without the mutant chains, e.g. because higher ER concentration of unfolded chains might cause nonspecific and therefore non-selective chain aggregation into gelatin-like structures [11].

Consistent with the significantly delayed procollagen clearance from the cells, Y-Arg and Gly substitutions cause similar ER dilation, indicating misfolded procollagen accumulation inside the ER (Fig 7) and possible cell stress. In agreement with other studies [11,50,51], the ER accumulation of misfolded procollagen does not trigger the conventional unfolded protein response (UPR) that is accompanied by upregulation of BIP expression and XBP1 splicing. An apparent increase in EIF2α phosphorylation, CHOP and αB-crystalline transcription, and decrease in calnexin observed here for Y-Arg and Gly substitutions (Fig 8) are consistent with the observations for cell stress response in osteoblasts from G610C mice [11], yet inconclusive. Our experience gained from the latter G610C mouse study suggests that conclusive evidence of cell stress response requires analysis of primary osteoblast cultures from multiple animals at low (preferably zero) passage. Dermal fibroblasts produce less collagen than osteoblasts, experience subtler ER dilation, and therefore less pronounced cell stress. The cell stress is particularly difficult to detect in human cells, since genetic variants unique for each subject might affect its severity.

Formation of a Cys766-S-S-Cys766 disulfide bond at the Gly substitution site improves procollagen folding and clearance from cells (Fig 6A and 6B), potentially reducing the severity of bone pathology. This bond might promote triple helix renucleation beyond the mutation site by keeping the three proα chains together. Formation of a Cys780-S-S-Cys780 bond at the Y-Arg substitution site has an opposite effect. This bond appears to inhibit triple helix renucleation, probably by disrupting the alignment of the chains. Reduced misfolding and accumulation of molecules with Gly-to-Cys substitution in two α1(I) chains due to disulfide bond formation might contribute to lower cell stress and therefore explain the surprising milder phenotype of homozygous vs. heterozygous mice with a Gly349-to-Cys substitution [52].

Note that Arg-to-Cys substitutions in the triple helical region of type II collagen were also reported to cause pathology [53,54]. In contrast to α1(I)-R780 mutations, conventional UPR and cell apoptosis were observed for α1(II)-R789C and α1(II)-R992C [55,56]. Similar to α1(I)-R780 mutations, α1(II)-R75C and α1(II)-R519C were found to cause some ER dilation without conventional UPR or apoptosis [56].

### Cell stress response to accumulation of misfolded procollagen might explain why Y- but not X-Arg substitutions cause bone pathology

Despite the inconclusive evidence for the cell stress response caused by Y-Arg substitutions, we believe that the idea of resulting osteoblast malfunction provides the most logical explanation for the following observations. Bone fragility and low mineral density were identified in a number of individuals with Cys for Y-Arg substitutions at positions 396, 858, 888, and 915 in the α1(I) chain of the triple helix [17,18,23]. In contrast, classical EDS [25] and Caffey disease [26] were seen with Cys for X-Arg substitutions at positions 134 and 836, respectively. Procollagen folding is expected to be significantly affected only by Arg substitution at Y-positions while ECM interactions of collagen should be affected by substitutions at both X- and Y-positions. Since decreased bone density and fractures are observed only with substitutions for Y- position Arg, this bone pathology is more likely to be related to osteoblast malfunction in response to abnormal procollagen folding than to direct effects of Arg to Cys substitutions on bone matrix and its mineralization. For the same reasons, skin and joint laxity as well arterial abnormalities are more likely to be related to aberrant interactions of Cys in the ECM. These EDS symptoms were reported in patients with both X- and Y-position Arg substitutions to Cys, which is not present in normal type I collagen.

Note that similarly mild bone pathology in patients with α1(I)-R780L and α1(I)-R780C substitutions is also more difficult to explain by disruptions of collagen interactions in the ECM. As expected, hydrophobic Leu and sulfhydryl Cys appear to have different effects on ECM composition and structure (Fig 9) but similar effects on procollagen folding (Fig 6) and ER dilation (Fig 7).

The idea of osteoblast malfunction as a major factor in bone pathology caused by procollagen misfolding has been discussed in several recent studies of dominant OI caused by Gly substitutions as well as recessive OI caused by mutations in procollagen chaperones [3,6,11,12,51,57–59]. It might contribute to the reported TGF-β signaling abnormalities in OI [11,60,61]. We think that the present analysis of Y-Arg substitutions provides further support for this idea, even though we have been able to study only patient fibroblasts.

## Supporting information

S1 File Representative Western blots. **Figure A**. Top panel. BIP and p-eIF2a were fluorescently labeled with Alexa Fluor 550 goat anti-rabbit IgG (red channel); eIF2a and β-actin were fluorescently labeled with Alexa Fluor 488 goat anti-mouse IgG (green channel). Magenta labels mark gel lanes from normal control (C5), α1(I)-G763S (763), α1(I)-G766C, α1(I)-G780C (780C), and α1(I)-G780L (780L) cells; the 811 sample was from a different study. Yellow molecular weight labels mark positions of the corresponding molecular weight standards, visualization of which requires contrast enhancement (70 kDa standard is visible as a faint red band at the left edge of the blot). Low intensity red p-eIF2a bands are masked by much higher intensity green eIF2a bands. Bottom panel. Contrast-enhanced red fluorescence channel shows thin, low intensity p-eIF2a bands and residual fluorescence of wide β-actin bands labeled with Alexa Fluor 488 (which is not completely eliminated by the Alexa Fluor 550 filter set). **Figure B**. Calnexin and calreticulin were fluorescently labeled with Alexa Fluor 550 goat anti-rabbit IgG (red channel); PDI and β-actin were fluorescently labeled with Alexa Fluor 488 goat anti-mouse IgG (green channel). ~85 kDa red bands (top panel) were identified as calnexin and ~55 kDa green bands (top panel) were identified as PDI. Magenta and yellow labels are the same as in Figure A, S1 File. **Figure C**. Procollagen α1(I) chain and β-actin were fluorescently labeled with Alexa Fluor 550 goat anti-rabbit IgG (red channel) and Alexa Fluor 488 goat anti-mouse IgG (green channel), respectively. Magenta and yellow labels are the same as in Figure A, S1 File.
(PDF)
