# Supplementary figures and images for: Substitutions for arginine at position 780 in triple helical domain of the α1(I) chain alter folding of the type I procollagen molecule and cause osteogenesis imperfecta
Source: PLoS One. 2018 Jul 10;13(7):e0200264. doi: 10.1371/journal.pone.0200264 (PMC6039012; doi:10.1371/journal.pone.0200264)

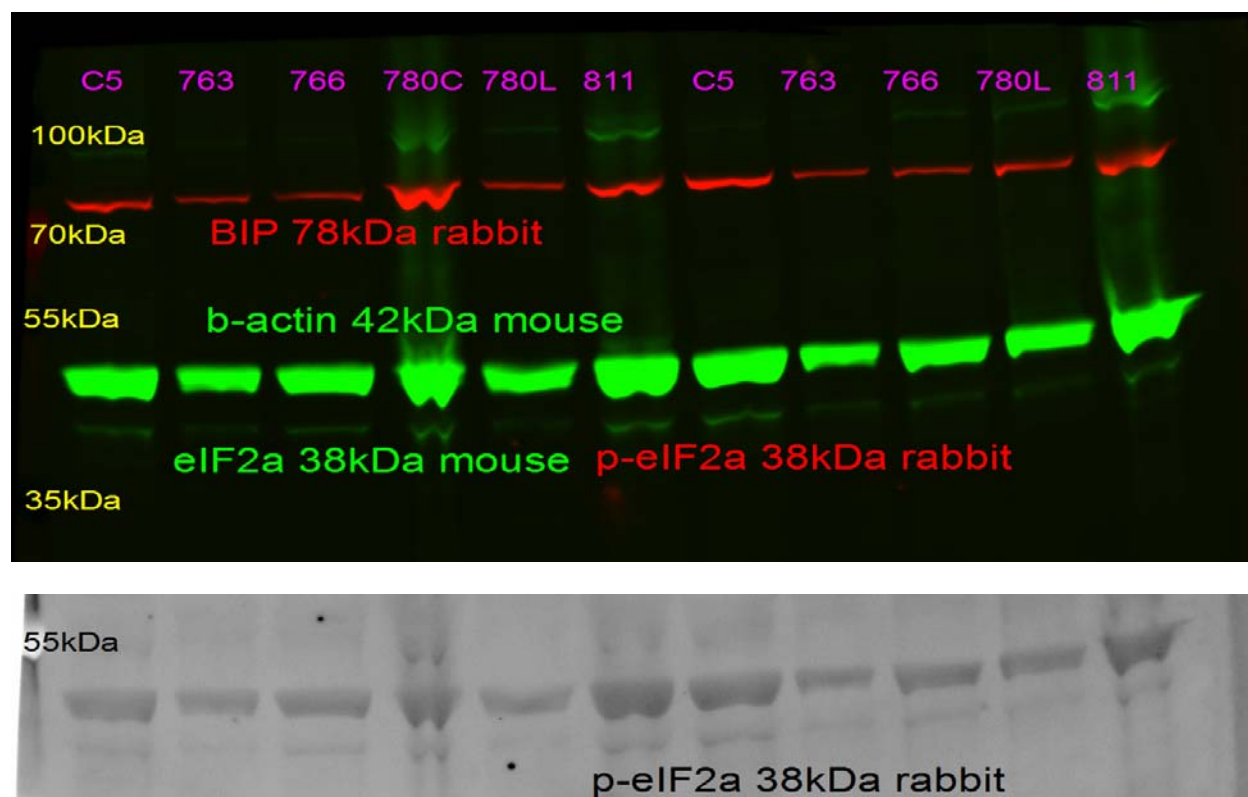

Figure A

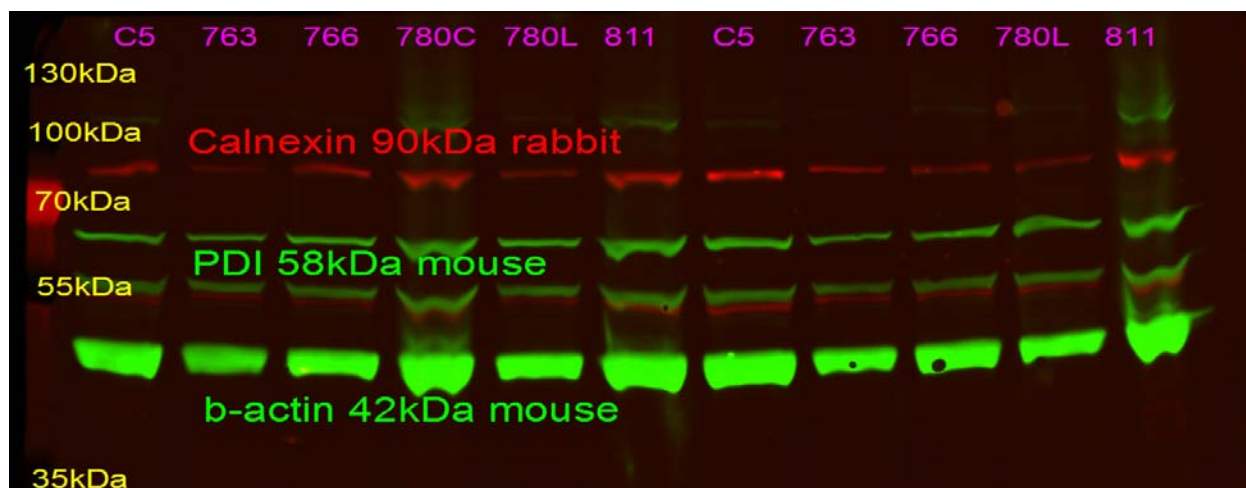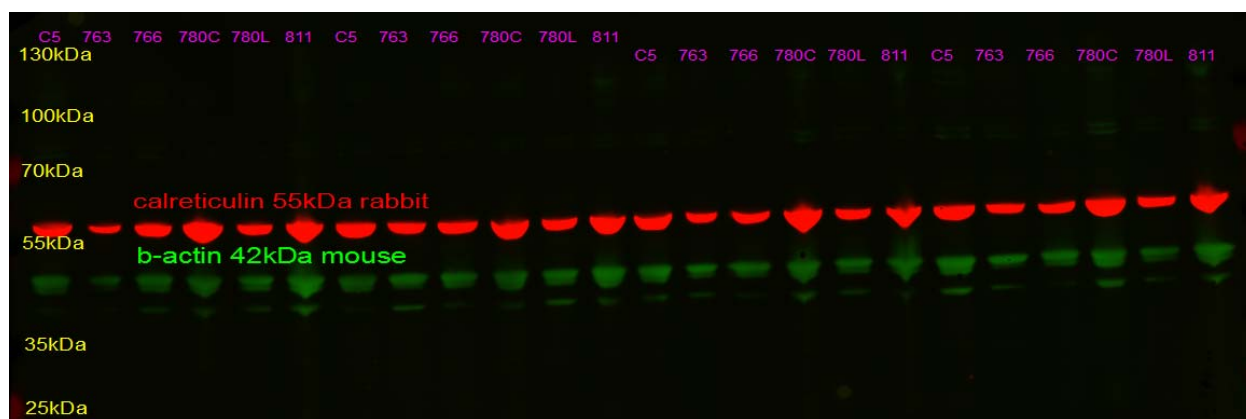

Figure B

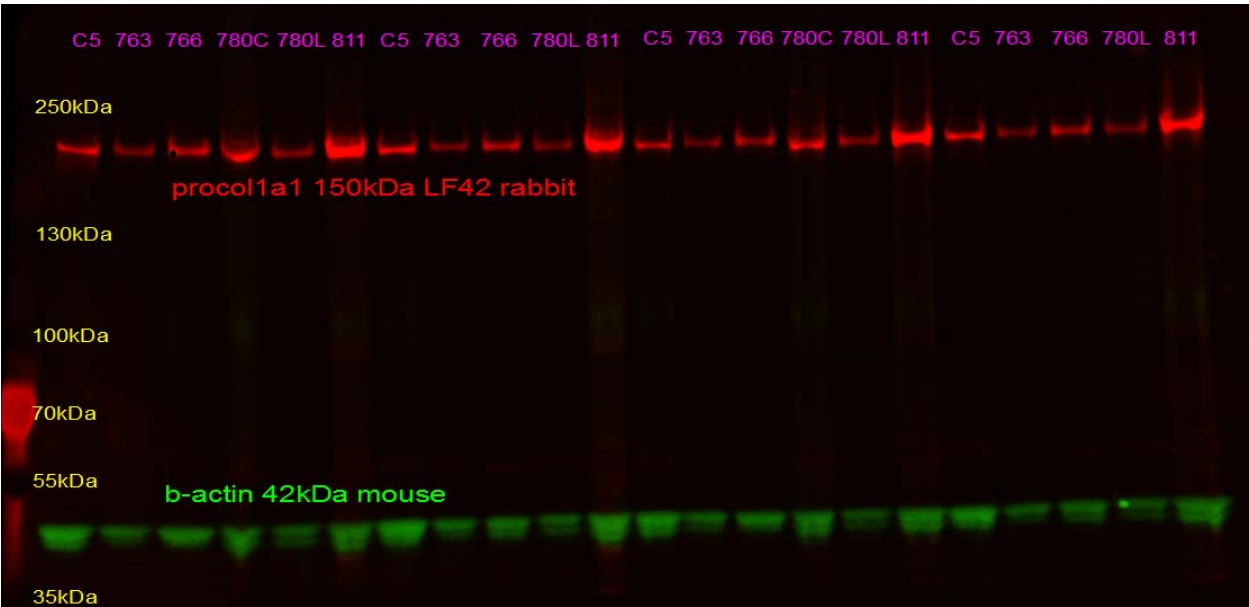

Figure C

Supplement: S1 File — Figure A. Top panel. BIP and p-eIF2a were fluorescently labeled with Alexa Fluor 550 goat anti-rabbit IgG (red channel); eIF2a and β-actin were fluorescently labeled with Alexa Fluor 488 goat anti-mouse IgG (green channel). Magenta labels mark gel lanes from normal control (C5), α1(I)-G763S (763), α1(I)-G766C, α1(I)-G780C (780C), and α1(I)-G780L (780L) cells; the 811 sample was from a different study. Yellow molecular weight labels mark positions of the corresponding molecular weight standards, visualization of which requires contrast enhancement (70 kDa standard is visible as a faint red band at the left edge of the blot). Low intensity red p-eIF2a bands are masked by much higher intensity green eIF2a bands. Bottom panel. Contrast-enhanced red fluorescence channel shows thin, low intensity p-eIF2a bands and residual fluorescence of wide β-actin bands labeled with Alexa Fluor 488 (which is not completely eliminated by the Alexa Fluor 550 filter set). Figure B. Calnexin and calreticulin were fluorescently labeled with Alexa Fluor 550 goat anti-rabbit IgG (red channel); PDI and β-actin were fluorescently labeled with Alexa Fluor 488 goat anti-mouse IgG (green channel). ~85 kDa red bands (top panel) were identified as calnexin and ~55 kDa green bands (top panel) were identified as PDI. Magenta and yellow labels are the same as in Figure A, S1 File. Figure C. Procollagen α1(I) chain and β-actin were fluorescently labeled with Alexa Fluor 550 goat anti-rabbit IgG (red channel) and Alexa Fluor 488 goat anti-mouse IgG (green channel), respectively. Magenta and yellow labels are the same as in Figure A, S1 File. (PDF) [file pone.0200264.s001.pdf]
